# Exposing Inequality: Environmental Injustice and Cardiovascular Health Disparities

**DOI:** 10.1016/j.jacadv.2025.101875

**Published:** 2025-06-20

**Authors:** Ritu Thamman, Samar A. Nasser, Keith C. Ferdinand, Sadeer Al-Kindi, Eric Brandt

**Affiliations:** a Division of Cardiology, University of Pittsburgh School of Medicine, Pittsburgh, Pennsylvania, USA; b Department of Clinical Research and Leadership, School of Medicine and Health Sciences, The George Washington University, Washington, District of Columbia, USA; c Section of Cardiology, John W. Deming Department of Medicine, Tulane University School of Medicine, New Orleans, Louisiana, USA; d Houston Methodist DeBakey Heart and Vascular Center, Weill Cornell Medical College, New York, New York, USA; e Division of Cardiovascular Medicine, University of Michigan Medical School, Ann Arbor, Michigan, USA

**Keywords:** air pollution, cardiovascular disease, environmental injustice, exposome, gene-environment interaction, social vulnerability

## Abstract

This review explores the intersection between environmental injustice and cardiovascular (CV) health disparities, highlighting how climate change, pollution, and environmental exposures disproportionately impact vulnerable populations. It delves into environmental racism, showing how non-Hispanic Black, Hispanic, and Native American communities face higher exposure to pollutants and climate-related hazards. This increased exposure contributes to greater CV morbidity and mortality, exacerbated by historical practices such as redlining and insufficient exposure regulations. The review points out the limitations of traditional CV risk models that overlook these environmental factors. Promoting transparency, community-driven solutions, and linking macro policies with local implementation are crucial to combating environmental injustice. It suggests that the emerging field of environmental cardiology can adopt eco-friendly sustainable practices and remote care solutions to reduce health care's carbon footprint, integrate environmental risks into prevention and treatment plans, and advocate for policies that reduce disparities in CV disease outcomes.

## Highlights

• Environmental risks drive cardiovascular disparities in underserved and racially minoritized communities.
• This review outlines how air pollution, heat, and toxins act as modifiable cardiovascular risks.
• Clinicians should integrate environmental exposure into CVD risk assessment and prevention strategies.
• Future efforts must embed environmental data into care, training, and equitable policy interventions.

Climate change portends one of the greatest threats to both planetary and human health, with multiexposure pathways that may progress the development of noncommunicable diseases, including cardiovascular disease (CVD).^1^ Climate and environmental risks are interconnected. The Planetary Boundaries Framework analyzes the boundaries between environmental risks and provides a comprehensive assessment of which planetary boundaries are crossed. This framework is now enhanced by data analytics, complex language modeling resulting in multilayered cause and effect loops that allow for more accurate predictions.^2^ For example, global warming impacts patients with CVD by leading to an intensification of the core temperature and affecting the circulation.^3^ The problem will intensify as 2023 was the hottest year recorded on earth, and this record is predicted to be surpassed in 2024.^4^ Similar to disparities in health across certain geographic areas and underserved populations, climate change poses a threat to population health with the greatest impact in specific communities. There is a 15-year life expectancy difference between adjacent zip codes from high vs low exposures to pollution and climate-related risk factors.^5^ Global warming and other climate change exposures disproportionately impact racial and ethnic populations and lower-wealth communities.^6^ In this State-of-the-Art review, we provide an overview of key environmental determinants relevant to cardiovascular (CV) medicine and discuss them in the context of inequities, environmental justice, and equity-driven solutions. See Central Illustration.

**Central Illustration:**
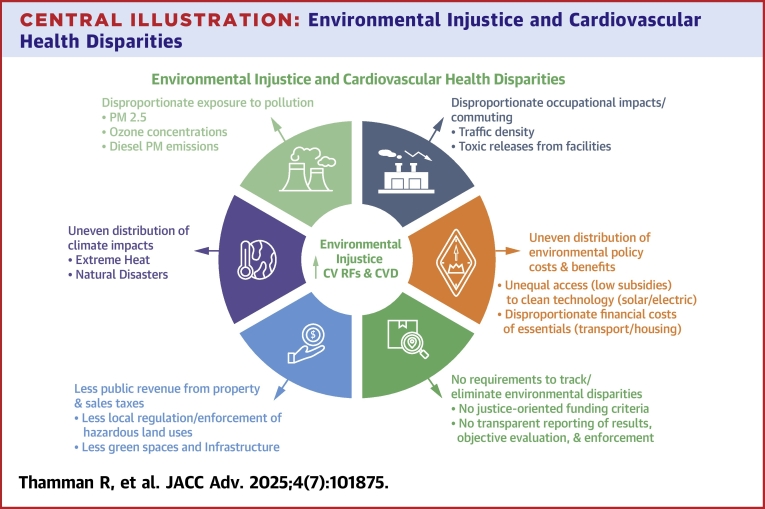
Environmental Injustice and Cardiovascular Health Disparities Environmental injustices that increase cardiovascular (CV) risk factors and cardiovascular disease (CVD).

## Traditional risk factors do not capture entire CVD risk, might be explained by environmental factors

The gene-environment interaction highlights how genetic predispositions and environmental exposures jointly shape disease risk and health outcomes. A person's genetic makeup can modify their response to environmental factors—such as toxins, stress, or diet—while these exposures can in turn influence how genes are expressed. Certain genotypes may heighten susceptibility to environmental risks (eg, toxins, pollutants), making some individuals more prone to disease than others. Environmental exposures can alter gene expression without changing DNA, called epigenetics, affecting biological pathways and long-term health. For example, air pollution may trigger disease in genetically vulnerable individuals or psychosocial stress can interact with genetic risk to influence conditions like CVD. Gene-environment interaction underscores the need for personalized prevention strategies that account for both biological and environmental factors.^7^

The INTERHEART (A Global Study of Risk Factors in Acute Myocardial Infarction) study included subjects from 52 high-, middle-, and low-income countries, with 9 modifiable risks factors accounting for 90% of the risk of having a first myocardial infarction (MI): smoking, dyslipidemia, hypertension, diabetes, abdominal obesity, psychosocial factors, consumption of fruits and vegetables, regular alcohol consumption, and physical inactivity.^8^ Importantly, 36% of the population-attributable risk of MI was accounted to smoking.^9^ However, while elevated plasma lipids, diabetes mellitus, hypertension, inflammation, and smoking are established causal pathways for atherothrombotic diseases, strategies to lower these known risk factors only partially mitigates risk. Notably, most patients with an acute CV event lack the established CV risk factor diagnoses.^9^ Thus, other factors that mediate CVD need to be understood in more detail. By 2030, approximately 75% of all deaths are expected to be caused by noncommunicable diseases.^10^ Part of these noncommunicable diseases is from the interplay between the genes of an individual and the environmental exposures.^11^ For example, while nearly half of U.S. adults have hypertension, only a quarter report achieving recommended levels of physical activity to help lower blood pressure, which is at least partially driven by the absence of green spaces to exercise.^12^ These environmental effects are noticeable since neighborhoods with greater environmental disadvantages have a higher prevalence of CVD and associated risk factors. Non-Hispanic Black (NHB) adults have the highest premature CV mortality rates in part due to their high prevalence of hypertension which is least twice any other race and ethnic population.^13^ Most of the U.S. population aged 18 to 44 years, NHB adults, and Hispanic individuals reside in places with high environmental burdens.^14^ Additionally, social vulnerability coexists and coinfluences the susceptibility to acquire CVD. Social Vulnerability Index, developed by the Centers for Disease Control and Prevention, differs from social determinants of health which encompasses economic stability, education, health care, neighborhood/built environment, community/social context, and food insecurity.^15^ Social Vulnerability Index measures a community's potential susceptibility to adverse effects resulting from natural or human-induced disasters. The burden of the community is measured by their vulnerability in 4 domains: socioeconomic status, household characteristics, racial and ethnic minority status, and housing. Beyond decreasing uncontrolled hypertension, obesity, diabetes, and physical inactivity, additional unrecognized causal mechanisms related to higher environmental burdens may be one of the reasons CVD mortality rates are increasing in the young population under the age of 50 years.^16^

## The exposome concept

Exposome, a word coined 2 decades ago, combines the words exposure and genome and describes the effect of environmental exposures on the genome.^17^ The exposome is the lifetime exposure of a population to their surrounding environment which encompasses both the built and natural environment along with social determinants of health and individual-level risk factors.^18^ The external exposome can change internal biochemical pathways. The exposome is a framework that integrates diffuse data from the environmental impact on CVD by measuring the exposures in the environment and then linking them to the internal biological responses which vary over time.^19^ This dynamic biologic response is hard to measure since it is time dependent but essential to assess its environmental impact on CVD. This requires the accurate and reliable measurement of many exposures along with their biological responses while simultaneously addressing the dynamic nature of the exposome.^20^ Studies show a strong correlation between the exposome and CV health, including higher CVD rates and CV risk factors related to the natural and built environment: air pollution, low green space, and toxic dumps, independent of low income or education levels.

## Disproportionate environmental risk factors in lower socioeconomic strata

From 2010 to 2017, the proportion of premature deaths in individuals aged 25-34 and 35-44 years rose, especially among NHB individuals and those individuals residing in rural areas.^21^ Two-thirds of all U.S. counties experienced increasing CVD rates among adults aged 35-64 years during 2010-2017 with high rates of uncontrolled hypertension and increasing prevalence of diabetes and obesity. In the last decade (post-2015), excess mortality rates for NHB compared with White populations widened, not only from violence and the COVID-19 pandemic but also from CVD, reversing decades of prior progress.^22^

Unequal exposure to detrimental environmental factors contributes to racial and ethnic disparities in CVD. For example, there is growing evidence of racial and ethnic inequalities in built environmental exposure and green space access.^23^ Also, the risk of air pollution exposure by particulate matter smaller than 2.5 μm (PM2.5) varies by income and race, and disparities in exposure relative to safety standards are increasing.^24^ NHB are 75% more likely to live in communities near high-emission or toxic industrial processes, and typically endure levels of air pollution at least 56% higher than what would be equitable.^25^

A study comparing pre- and post-COVID-19 pandemic, air quality found larger air pollution exposure among Hispanic and Asian communities.^26^ This study highlights the need for robust and accurate air quality data collections in underserved communities since communities of color are disproportionately impacted by the effects of climate change, such as heat waves and droughts, which are exacerbated by emissions from power plants and other industrial sources.^27^ These communities often face higher exposure to environmental risks and have fewer resources to adapt to these challenges.^28^

## Disparities in environmental CVD risk

Independent of social factors, environmental exposure contributes to the prevalence of CVD and its risks. Recently, a study found that increases in environmental burden were associated with progressive increases in the prevalence of CVD. Using the Centers for Disease Control Population-Level Analysis and Community Estimates, the Socio-environmental Justice Index was divided into quartiles from the least vulnerable to the most vulnerable census tracts. Compared to least, the most vulnerable had a higher rate of coronary artery disease, stroke, and CV risk factors.^29^ Since the majority of NHB and Hispanic individuals reside in places with significant environmental burdens, the role of environmental factors must be addressed to obtain the most accurate information regarding individual patients' environmental risk factors. This information should be used to target these vulnerable patients for early prevention and screening and advocating for policies that will allocate funds to treat the higher rate of CVD and CVD risk factors.

## Air pollution

There are 2 main forms of air pollution: PM2.5 and household air pollution from the use of solid fuels like coal, wood, agricultural waste for cooking, mostly found in low-income countries. Air pollution causes 1-in-5 deaths worldwide, equivalent to the number caused by smoking. Air quality regulations that lower ambient PM2.5 levels reduce morbidity and mortality.^30^ PM2.5 and nitrogen dioxide (NO2) are harmful air pollutants associated with CVDs especially the PM2.5 particles which are small enough to be inhaled into the respiratory system and penetrate the bloodstream.^31^ A UK Biobank study found that long-term exposures to PM2.5 and NO2 were associated with elevated risks cardiometabolic multimorbidity and death.^32^ Those with lower economic levels were more susceptible to air pollution–related cardiometabolic multimorbidity. One major source of PM2.5 pollution is nitrogen oxides from vehicle emissions. Hispanic and Asian people living near semitrailer traffic at giant warehouses have 2.5 times the nationwide average of NO2 and air pollution.^33^ More garbage dumps, industrial plants, congested roads, and airports exist in neighborhoods of color, leading to higher exposure to these pollutants. There is an independent association between short-term exposure to particulate matter of PM10 and PM2.5 and all-cause and CV mortality in over 600 cities across the world.^34^ The Global Burden of Disease Study shows that CVD accounts for the majority (≈60%) of the excess in morbidity and mortality due to PM2.5.^35^ Higher long-term PM2.5 exposure is associated with impaired plaque healing in patients with recurrent acute coronary syndrome, and with the presence of more vulnerable plaque features at the culprit lesion.^36^ Patients at the highest risk from air pollution are those with atherosclerotic CVD and recent hospitalization for an acute coronary syndrome or heart failure.^37^ Long-term exposure to PM2.5 and NO2 is associated with abdominal aortic aneurysms, even when air pollution concentrations are below the recommended air quality thresholds established by the World Health Organization of 5 μg/m^3^.^38^

For every 10 μg/m^3^ increase in PM2.5, there was a 12% increased risk of incident acute MI, a 21% increased risk of ischemic heart disease death, and an 8% increased risk of CVD death in a California cohort.^39^ Newer models suggest that the shape of the exposure-response curve is nonlinear, so emission reductions at all levels of air pollution will decrease the number of attributable deaths.^40^ The Environmental Protection Agency (EPA) tightened the Air Quality Index limit from 12.0 μg/m^3^ to 9.0 μg/m^3^ in May 2024 setting a stronger emissions standard for cars and light trucks.^41^ Tailoring Air Quality Index guidelines to CVD risk could be more efficient since a high number of events treated is needed to reduce CVD from PM25.^42^

## Ozone

Ozone (O3), the primary air pollutant in photochemical smog, is a global public health concern as it adversely affects CV health. O3 continues to rise globally, parallel to the mounting burden of CVD. O3 increases hospitalizations for CVD, and specific populations display heightened vulnerability to these effects.^43^

In a prospective study of approximately 3.2 million Chinese participants, there was a significant influence of long-term O3 exposure on CVD mortality; a 10.0 μg/m^3^ increase in the O3 level above 87 μg/m^3^ had a 13.9% and 25.0% higher risk of CVD and ischemic heart disease mortality, respectively.^44^ Notably, farmers and individuals with lower education levels had a higher risk of CVD mortality. At the same time, diabetes, hypertension, dyslipidemia, general obesity, and abdominal obesity mediated greater than half of the association between O3 exposure and CVD mortality. More exposure-response studies could inform future air quality standard revisions and environmental health impact assessments for the populations living in dense O3 areas of the world.

## Natural disasters

The impact of natural disasters continues to rise affecting both mental and physical outcomes contributing to CVD. The pathological CV effects of mental stress are mediated through the neuroendocrine-immune axis to the vasculature leading to endothelial dysfunction and vascular inflammation.^45^ Mental stress stimulates increases in blood pressure, while natural disasters damage the community health care infrastructure, resulting in difficulty accessing emergency services, medications, and devices. On August 29, 2005, Hurricane Katrina led to a subsequent elevation in CVD among older adults in Orleans Parish.^46^ Additionally, CVD disparities were exacerbated between NHB and White older adults during and following landfall.

## Extreme heat

Extreme heat links to CVD via dehydration, hemoconcentration, hypercoagulability, sympathetic activation, and inflammatory mediators. A 1 °C increase in temperature increased CVD-related mortality from stroke and coronary heart disease by 2.1%.^47^ The extreme heat risk has been shown to be higher in vulnerable subgroups, including older people, people with preexisting conditions, and the socioeconomically deprived. According to Singh et al there are temporal changes in heat-related CVD deaths as well as the interactive effect of heat with other environmental factors. Given the rising climate change issues, they advocate for future studies focusing on lower-middle-income countries and vulnerable populations.^48^

Since a major source of PM2.5 pollution is nitrogen oxides from vehicle emissions, proximity to industry, high traffic areas, and less greenspace leads to more exposure to air pollution and extreme heat, increasing their impact in these disadvantaged populations.^49^ Lower levels of tree canopy and higher urban heat by as much as 7 °C are present in formerly redlined areas relative to their nonredlined neighbors.^50^ Land Surface Temperature Data show patterns of surface urban heat disparities in >1,000 counties across the United States with daytime urban warming greater in lower-income census tracts.^51^ Neighborhoods with more greenspace and greater walkability housed populations with lower CVD mortality compared with areas without greenspace and walkability.^52^ Communities of color are less likely to have green spaces and recreational facilities that create opportunities for physical activity.^53^ The absence of a green canopy is linked to negative heat effects of climate change, resulting in a higher ozone level.^54^ Evaluating exposures should include assessing ambient air pollution based on the ZIP code, levels of solid fuel combustion, in-home ventilation, time spent in traffic, and if they reside close to highways.^55^

## Heavy metals

Exposure to lead, cadmium, or arsenic is associated with CV death mostly from ischemic heart disease since these contaminants can displace or mimic essential divalent cations and disrupt key cellular functions.^56^ Heavy metal contamination is found more in urban soil in low-income and/or predominantly minority communities.^57^ There is an association between urinary metal levels and coronary artery calcification progression over 10 years.^58^ Recently, a study in diabetic patients with prior MIs did not show any reduction in CV events with chelation therapy, despite a reduction in lead levels.^59^

## Microplastics

Microplastics (MNPs) are solid plastic particles ≤5 mm in size. MNPs are pervasive in the environment, including food and drinks, and have negative health effects.^60^ For example, patients with carotid artery plaque with MNPs had a higher risk of MI, stroke, or death from any cause than those in whom MNPs were not detected.^61^ Low-income and Black communities are disproportionately affected by MNPs, by living nearer to oil refineries and contaminated water supplies.^62^

## Initiatives addressing environmental injustice

The 2 main policies for environmental justice are the Inflation Reduction Act (IRA) and the Justice40 program. The IRA became law in 2022 with approximately $369 billion appropriated with tax incentives for environmental justice. The IRA intends to improve indoor air quality in schools in low-income communities, replacing diesel-fueled garbage trucks and city buses with zero-emission vehicles, and creating pollution-reducing technologies in disadvantaged communities to reduce legacy pollution, increase access to clean energy and create jobs for disadvantaged communities.^63^ The IRA was expected to catalyze actions in both the public and private sectors. For example, the California Air Resources Board recently approved the Advanced Clean Cars II rule, which mandates that by 2035 all new cars, trucks, and SUVs sold in California must be zero-emission vehicles.^64^ As federal agencies distribute these billions of dollars, transparency to track how those funds are spent, and whether communities can access them to implement community-driven climate change solutions will be key to IRA's environmental justice goals. In addition, the IRA states that greenhouse gases are air pollutants for the first time to help catalyze the transition to clean energy.^65^ The Justice 40 Initiative, an Executive Order, mandates that at least 40% of federal investments in climate policy flow to historically disadvantaged communities.^66^ It is not clear how “40% of benefits” is defined or should be measured. Business leaders, community-based organizations, and environmental justice groups may not align on what is a “benefit.” Reimbursement policy, incentive structures, community engagement models, and technology-driven solutions need to be addressed. Additionally, linking macro policies at the federal level with regional local/state driven policy may be an important step to achieve environmental justice.

## Impact of policies

Race and ethnicity are the biggest risk factor for environmental exposure regardless of which state of residence, urban or rural areas, or income levels.^67^ NHB populations are 75% more likely to live in high-emission or toxic industrial areas, and face double the level of air pollution than others.^68^ Redlining, a practice to give the worst grades to deny mortgages by the federally backed Home-Owners Loan Corporation in the 1930s, and segregation have contributed to higher levels of exposures for racial populations. For example, redlined communities have double the oil well density as nonredlined communities.^69^ Redlining perpetuates a de facto residential segregation thereby creating neighborhoods predominantly of people of color with low household income contributing to persistent systemic racism.^70^ In addition, less stringent planning regulations on Native American lands have enabled waste dumps and uranium mining to proliferate and raise Native American exposures.^71^ These data only include pollution from natural gas facilities and oil production facilities, not pollution from petroleum refineries which would detect greater negative environmental health impacts.

## Clinical implementation of environmental considerations in cardiovascular risk assessment and prevention

The future of environmental cardiology—training, practice, research, and advocacy—in the fight against climate change is evolving. Environmental sustainability in high resource cardiology settings are key to reducing the carbon footprint in the cardiac catheterization laboratory, pacemaker monitoring, and in-hospital care including cardiac surgery.^72^ Despite other advanced imaging modalities, echocardiography is the greenest, with the least carbon footprint, just 1% to 20% of that of cardiac magnetic resonance or single-photon emission tomography.^73^ Remote monitoring of pacemakers, telephone consultations over in-person evaluation, virtual cardiology conferences over traditional in-person conferences will reduce the carbon footprint in cardiology.^74^

Policies to protect against environmental pollution are needed to improve CV health outcomes. Physicians, especially CV specialists and other health care providers, and public health officials have a shared responsibility to advocate for decarbonization and urge health care systems to aim for a net zero carbon footprint.^75^ However, getting to net zero carbon footprint will take time, require lowering fossil fuels usage, decreasing carbon-intensive supply chains of pharmaceuticals and procedures, and divesting from carbon-intensive industries.^76^

In the meantime, solutions can be initiated. For example, air pollution maps of higher exposure could be used to target CVD prevention by addressing other CVD risk factors earlier.^77^ Linking decarbonization with economic programs, such as affordable housing, higher wages, and educational and job opportunities may elevate public support for climate change mitigation in communities of color. Prescribing high-efficiency particulate air filters that reduce mean diastolic blood pressure and systolic blood pressure may reduce hypertension in those living with environmentally disparities.^78^

Clinicians can integrate brief environmental exposure histories—focused on air pollution, noise, and extreme heat—into intake forms or electronic medical records templates to identify high-risk individuals, particularly those with existing CVD or living in vulnerable areas.^79^ Tools like the *EPA's EJScreen*, *Centers for Disease Control and Prevention's Environmental Public Health Tracking*, or ZIP code–based indices of pollution burden can inform personalized risk assessments.^80^ Simple checklists on housing, occupation, and proximity to pollution sources may aid screening. Environmental risks should be treated as modifiable CVD risk factors. For example, clinicians can advise limiting outdoor activity during high pollution days, support use of air purifiers, and consider exposure in risk stratification alongside traditional metrics. Training should include curricula on environmental determinants of CV health, case-based learning, and practical tools to assess and counsel on exposures.^81^ Embedding this within preventive cardiology and population health rotations is essential. Models like the *National Academy of Medicine's Framework for Integrating Social and Environmental Determinants into Care* and the *Clinical Climate and Health Framework* provide guidance on embedding environmental data into clinical workflows.^80^ Partnering with local public health departments and community organizations can enhance trust, facilitate targeted interventions, and codevelop culturally relevant solutions for exposure mitigation and advocacy.^80^

## Conclusions

The mechanisms by which CVD develops are multifactorial and extend beyond traditional risk factors and into the exposome. Understanding, evaluating, and constructing policy changes to modify these environment factors and their potential adverse outcomes can be impactful in the drive to reduce incident CVD.

Thus, the time is ripe for all cardiologists to treat environmental injustice as a cardiac risk factor to reduce CV health disparities and improve outcomes for the global population.^82^

## Funding support and author disclosures

The authors have reported that they have no relationships relevant to the contents of this paper to disclose.

## References

[1] Braunwald E. (2024). CV effects of climate change. Eur Heart J.

[2] Richardson K., Steffen W., Lucht W. (2023). Earth beyond six of nine planetary boundaries. Sci Adv.

[3] Desai Y., Khraishah H., Alahmad B. (2023). Heat and the heart. Yale J Biol Med.

[4] Esper J., Torbenson M., Büntgen U. (2024). 2023 summer warmth unparalleled over the past 2,000 years. Nature.

[5] Rajagopalan S., Ramaswami A., Bhatnagar A. (2024). Toward heart-healthy and sustainable cities: a policy statement from the AHA. Circulation.

[6] Kazi D.S., Katznelson E., Liu C.L. (2024). Climate change and CV health: a systematic review. JAMA Cardiol.

[7] Virolainen S.J., VonHandorf A., Viel K.C.M.F., Weirauch M.T., Kottyan L.C. (2023). Gene-environment interactions and their impact on human health. Genes Immun.

[8] Yusuf S., Hawken S., Ounpuu S. (2004). Effect of potentially modifiable risk factors associated with myocardial infarction in 52 countries: case-control study. Lancet.

[9] Grundy S.M., Stone N.J., Bailey A.L. (2019). 2018 AHA/ACC guideline on the management of blood cholesterol: a report of the ACC/AHA Task Force on clinical practice guidelines. J Am Coll Cardiol.

[10] Martin S.S., Aday A.W., Almarzooq Z.I. (2024). 2024 heart disease and stroke statistics: a report of US and global data from the AHA. Circulation.

[11] Aggarwal R., Yeh R.W., Joynt Maddox K.E. (2023). CV risk factor prevalence, treatment, and control in US adults aged 20 to 44 years, 2009 to March 2020. JAMA.

[12] Liu M., Patel V.R., Salas R.N. (2024). Neighborhood environmental burden and CV health in the US. JAMA Cardiol.

[13] Malhi J.K., McEvoy J.W., Blumenthal R.S., Jacobsen A.P. (2024). Climate change and CV health: recent updates and actions for healthcare. Am Heart J Plus.

[14] Wang Y., Jinfeng W. (2020). Modelling and prediction of global non-communicable diseases. BMC Public Health.

[15] Ibrahim R., Sainbayar E., Pham H.N. (2024). Social vulnerability index and cardiovascular disease care continuum: a scoping review. JACC Adv.

[16] GBD 2019 Risk Factors Collaborators (2020). Global burden of 87 risk factors in 204 countries and territories, 1990-2019: a systematic analysis for the GBD Study 2019. Lancet.

[17] Wild C.P. (2005). Complementing the genome with an “exposome”: the outstanding challenge of environmental exposure measurement in molecular epidemiology. Cancer Epidemiol Biomarkers Prev.

[18] Motairek I., Makhlouf M.H.E., Rajagopalan S., Al-Kindi S. (2023). The exposome and cardiovascular health. Can J Cardiol.

[19] Münzel T., Sørensen M., Hahad O. (2023). The contribution of the exposome to the burden of CV disease. Nat Rev Cardiol.

[20] Vrijheid M. (2014). The exposome: a new paradigm to study the impact of environment on health. Thorax.

[21] Berberian A.G., Gonzalez D.J.X., Cushing L.J. (2022). Racial disparities in climate change-related health effects in the US. Curr Environ Health Rep.

[22] Kobo O., Misra S., Banerjee A. (2024). Post-COVID changes and disparities in cardiovascular mortality rates in the United States. Prev Med Rep.

[23] Arun A.S., Caraballo C., Sawano M. (2024). Cause-specific mortality rates among the US Black population. JAMA Netw Open.

[24] Jbaily A., Zhou X., Liu J. (2022). Air pollution exposure disparities across US population and income groups. Nature.

[25] Clark L.P., Millet D.B., Marshall J.D. (2014). National patterns in environmental injustice and inequality: outdoor NO2 air pollution in the US. PLoS One.

[26] Bluhm R., Polonik P., Hemes K.S. (2022). Disparate air pollution reductions during California's COVID-19 economic shutdown. Nat Sustain.

[27] Montague D. (2022). Systemic environmental racism exposed. Nat Sustain.

[28] Zeighami A., Kern J., Yates A.J. (2023). U.S. West Coast droughts and heat waves exacerbate pollution inequality and can evade emission control policies. Nat Commun.

[29] Khadke S., Kumar A., Al-Kindi S. (2024). Association of environmental injustice and CVD and risk factors in the United States. J Am Heart Assoc.

[30] Rajagopalan S., Brauer M., Bhatnagar A. (2020). Personal-level protective actions against particulate matter air pollution exposure: a scientific statement from the AHA. Circulation.

[31] Bhatnagar A. (2017). Environmental determinants of CVD. Circ Res.

[32] Luo H., Zhang Q., Yu (2022). Long-term exposure to ambient air pollution is a risk factor for trajectory of cardiometabolic multimorbidity: a prospective study in the UK Biobank. EBioMedicine.

[33] Kerr G.H., Meyer M., Goldberg D.L. (2024). Air pollution impacts from warehousing in the US uncovered with satellite data. Nat Commun.

[34] Liu C., Chen R., Sera F. (2019). Ambient particulate air pollution and daily mortality in 652 cities. N Engl J Med.

[35] Roth G.A., Mensah G.A., Johnson C.O. (2020). Global burden of CVD and risk factors, 1990- 2019: update from the GBD 2019 study. J Am Coll Cardiol.

[36] Russo M., Rinaldi R., Camilli M. (2023). Air pollution and plaque healing in acute coronary syndromes. Eur Heart J.

[37] Langenbach M.C., Mayrhofer T., Langenbach I. (2023). Association between mall particulate matter and obstructive coronary artery disease in patients with stable chest pain across the US: insights from the PROMISE trial. J Cardiovasc Comput Tomogr.

[38] Hahad O., Daiber A., Münzel T. (2024). Breathing danger: linking air pollution to CVD and increased risk of abdominal aortic aneurysm. Eur Heart J.

[39] Alexeeff S.E., Deosaransingh K., Van Den Eeden S. (2023). Association of long-term exposure to particulate air pollution with CV events in California. JAMA Netw Open.

[40] Lelieveld J., Haines A., Burnett R. (2023). Air pollution deaths attributable to fossil fuels: observational and modelling study. BMJ.

[41] Henning R.J. (2024). Particulate matter air pollution is a significant risk factor for CVD. Curr Probl Cardiol.

[42] Brook R.D., Rajagopalan S., Al-Kindi S. (2024). Public health relevance of US EPA air quality index activity recommendations. JAMA Netw Open.

[43] Jiang Y., Huang J., Li G. (2023). O3 pollution and hospital admissions for CV events. Eur Heart J.

[44] Zenglei Z., Wang A., Lin C. (2024). Association of long-term exposure to O3 with CV mortality and its metabolic mediators: evidence from a nationwide, population-based, prospective cohort study. Lancet Reg Health West Pac.

[45] Jaskanwal D.S.S., Toya T., Ahmad A. (2022). Mental stress and its effects on vascular health. Mayo Clinic Proc.

[46] Becquart N.A., Naumova E.N., Singh G., Chui K.K.H. (2018). CVD hospitalizations in Louisiana Parishes' elderly before, during and after Hurricane Katrina. IJERPH.

[47] Liu J., Varghese B.M., Hansen A. (2022). Heat exposure and CV health outcomes: a systematic review and meta-analysis. Lancet Planet Health.

[48] Singh N., Areal A.T., Breitner S. (2024). Heat and CV mortality: an epidemiological perspective. Circ Res.

[49] Rajagopalan S., Al-Kindi S.G., Brook R.D. (2018). Air pollution and CVD: JACC State-of-the-Art Review. J Am Coll Cardio.

[50] Hoffman J.S., Shandas V., Pendleton N. (2020). The effects of historical housing policies on resident exposure to intra-urban heat: a study of 108 US urban areas. Climate.

[51] Khatana S.A.M., Werner R.M., Groeneveld P.W. (2022). Association of extreme heat and CV mortality in the US: a county-level longitudinal analysis from 2008 to 2017. Circulation.

[52] Liao N.S., Van Den Eeden S.K., Sidney S. (2022). Joint associations between neighborhood walkability, greenness, and particulate air pollution on CV mortality among adults with a history of stroke or acute myocardial infarction. Environ Epidemiol.

[53] Mohai P., Lantz P.M., Morenoff J. (2009). Racial and socioeconomic disparities in residential proximity to polluting industrial facilities: evidence from the Americans' Changing Lives Study. Am J Public Health.

[54] Zhang K., Brook R.D., Li Y. (2023). Air pollution, built environment, and early CVD. Circ Res.

[55] Brauer M., Casadei B., Harrington R.A. (2021). Taking a stand against air pollution-the impact on CVD: a joint opinion from the World Heart Federation, ACC, AHA, and the European Society of Cardiology. Circulation.

[56] Lamas G.A., Bhatnagar A., Jones M.R. (2023). Contaminant metals as CV risk factors: a scientific statement from the AHA. J Am Heart Assoc.

[57] Jones D.J., Yu X., Guo Q. (2022). Racial disparities in the Heavy metal contamination of urban soil in the Southeastern US. Int J Environ Res Public Health.

[58] McGraw K.E., Schilling K., Glabonjat R.A. (2024). Urinary metal levels and coronary artery calcification: longitudinal evidence in the multi-ethnic study of atherosclerosis. J Am Coll Cardiol.

[59] Lamas G.A., Anstrom K.J., Navas-Acien A. (2024). Edetate disodium-based chelation for patients with a previous myocardial infarction and diabetes: TACT2 randomized clinical trial. JAMA.

[60] Thompson R.C., Courtene-Jones W., Boucher J. (2024). Twenty years of microplastics pollution research-what have we learned?. Science.

[61] Marfella R., Prattichizzo F., Sardu C. (2024). Microplastics and nanoplastics in atheromas and CV events. N Engl J Med.

[62] United Nations Report (2021). https://www.unep.org/resources/report/neglected-environmental-justice-impacts-marine-litter-and-plastic-pollution.

[63] Glicksman R.L. (2023). Protecting the public health with the inflation reduction act - provisions affecting climate change and its health effects. N Engl J Med.

[64] Yu Q., He B.Y., Ma J. (2023). California's zero-emission vehicle adoption brings air quality benefits, yet equity gaps persist. Nat Commun.

[65] Cha M.J., Pastor M. (2022). Just transition: framing, organizing, and power-building for decarbonization. Energy Res Soc Sci.

[66] Morello-Frosch R., Osagie K.O. (2023). The climate gap and the color line - racial health inequities and climate change. N Engl J Med.

[67] Motairek I., Lee E.Y., Janus S. (2022). Historical neighborhood redlining and contemporary cardiometabolic risk. J Am Coll Cardiol.

[68] Lane H.M., Morello-Frosch R., Marshall J. (2022). Historical redlining is associated with present-day air pollution disparities in U.S. Cities. Environ Sci Technol Lett.

[69] Gonzalez D.J.X., Nardone A., Nguyen A.V. (2023). Historic redlining and the siting of oil and gas wells in the United States. J Expo Sci Environ Epidemiol.

[70] Al-Kindi S., Motairek I., Kreatsoulas C. (2023). Historical neighborhood redlining and CV risk in patients with CKD. Circulation.

[71] Albert M.A., Churchwell K., Desai N. (2024). Addressing structural racism through public policy advocacy: a policy statement from the AHA. Circulation.

[72] Tessum C.W., Paolella D.A., Chambliss S.E. (2021). PM_2.5_ polluters disproportionately and systemically affect people of color in the US. Sci Adv.

[73] Gunasekaran S., Szava-Kovats A., Battey T. (2024). CV imaging, climate change, and environmental sustainability. Radiol Cardiothorac Imaging.

[74] Barratt A.L., Li Y., Gooroovadoo I. (2023). Environmental impact of CV healthcare. Open Heart.

[75] Singh H., Eckelman M., Berwick D.M. (2022). Mandatory reporting of emissions to achieve net-zero health care. N Engl J Med.

[76] Sehgal A.R. (2023). Climate change and health system financial investments. J Gen Intern Med.

[77] Pastor M., Cha M.J., Méndez M. (2024). California dreaming: why environmental justice is integral to the success of climate change policy. Proc Natl Acad Sci U S A.

[78] Young M.T., Jansen K., Cosselman K.E. (2023). Blood pressure effect of traffic-related air pollution: a crossover trial of in-vehicle filtration. Ann Intern Med.

[79] Sorensen C.J., Fried L.P. (2024). Defining roles and responsibilities of the health workforce to respond to the climate crisis. JAMA Network Open.

[80] Ganatra S., Khadke S., Kumar A. (2024). Standardizing social determinants of health data: a proposal for a comprehensive screening tool to address health equity a systematic review. Health Aff Sch.

[81] Khraishah H., Ganatra S., Al-Kindi S.G. (2023). Climate change, environmental pollution, and the role of cardiologists of the future. J Am Coll Cardiol.

[82] Brandt E.J., Tobb K., Cambron J.C. (2023). Assessing and addressing social determinants of CV health: JACC State-of-the-Art Review. J Am Coll Cardiol.

